# Usability of a Patient Education and Motivation Tool Using Heuristic Evaluation

**DOI:** 10.2196/jmir.1244

**Published:** 2009-11-06

**Authors:** Ashish Joshi, Mohit Arora, Liwei Dai, Kathleen Price, Lisa Vizer, Andrew Sears

**Affiliations:** ^2^Usability Engineering GroupXerox CorporationBaltimoreMDUSA; ^1^Department of Information SystemsUniversity of MarylandBaltimore CountyBaltimoreMDUSA

**Keywords:** Computers, health, education, usability, heuristic

## Abstract

**Background:**

Computer-mediated educational applications can provide a self-paced, interactive environment to deliver educational content to individuals about their health condition. These programs have been used to deliver health-related information about a variety of topics, including breast cancer screening, asthma management, and injury prevention. We have designed the Patient Education and Motivation Tool (PEMT), an interactive computer-based educational program based on behavioral, cognitive, and humanistic learning theories. The tool is designed to educate users and has three key components: screening, learning, and evaluation.

**Objective:**

The objective of this tutorial is to illustrate a heuristic evaluation using a computer-based patient education program (PEMT) as a case study. The aims were to improve the usability of PEMT through heuristic evaluation of the interface; to report the results of these usability evaluations; to make changes based on the findings of the usability experts; and to describe the benefits and limitations of applying usability evaluations to PEMT.

**Methods:**

PEMT was evaluated by three usability experts using Nielsen’s usability heuristics while reviewing the interface to produce a list of heuristic violations with severity ratings. The violations were sorted by heuristic and ordered from most to least severe within each heuristic.

**Results:**

A total of 127 violations were identified with a median severity of 3 (range 0 to 4 with 0 = no problem to 4 = catastrophic problem). Results showed 13 violations for visibility (median severity = 2), 38 violations for match between system and real world (median severity = 2), 6 violations for user control and freedom (median severity = 3), 34 violations for consistency and standards (median severity = 2), 11 violations for error severity (median severity = 3), 1 violation for recognition and control (median severity = 3), 7 violations for flexibility and efficiency (median severity = 2), 9 violations for aesthetic and minimalist design (median severity = 2), 4 violations for help users recognize, diagnose, and recover from errors (median severity = 3), and 4 violations for help and documentation (median severity = 4).

**Conclusion:**

We describe the heuristic evaluation method employed to assess the usability of PEMT, a method which uncovers heuristic violations in the interface design in a quick and efficient manner. Bringing together usability experts and health professionals to evaluate a computer-mediated patient education program can help to identify problems in a timely manner. This makes this method particularly well suited to the iterative design process when developing other computer-mediated health education programs. Heuristic evaluations provided a means to assess the user interface of PEMT.

## Introduction

Computer technology has been widely used for education of both patients and health care professionals. Patient receptivity to computerized education is reported to be high across diverse medical settings and age groups. Computerized patient education has also been shown to increase patient knowledge, but little is reported about the results of findings of usability assessments of computerized patient education programs.

The objective of this case study was to describe the usability of the Patient Education and Motivation Tool (PEMT) through heuristic evaluation of the interface. We report the results of the usability evaluation, make changes based on the findings of the usability experts, and describe the benefits and limitations of applying usability evaluations to PEMT. We used Nielson’s 10 usability heuristics [6] to identify potential usability problems, describe severity ratings for each heuristic violation, and use the results to improve the overall usability of PEMT. This paper presents the results of the heuristic evaluation of PEMT, system changes performed based on the evaluation, and planned future research.

### Human-Computer Interaction Evaluation

Human factor or usability engineering is a discipline that investigates human/machine interface issues, using a wide array of methodologies [7]. These methodologies vary in terms of research design, complexity, cost, duration, and relevance to operational programs [7]. The two approaches for evaluating the human-computer interaction (HCI) characteristics of a system include inspection methods or user evaluations [5,8]. Inspection methods are based on reviews of a system, often by experts, which can be guided by usability heuristics, user tasks, or other information [5,8,9]. User evaluations measure user task performance in a lab setting [5,8]. Using these methods in system development has been recognized as an important way to ensure the usability of the end product [5,8,9].

Nielson defines heuristic evaluation as a measurement that utilizes heuristics in order to find usability problems [4]. Nielson’s method uses a small set of principles, guidelines, or heuristics that are systematically assessed against a target system in order to identify problems and their severity, as well consequences for the user [4,7]. Heuristic evaluation is an effective usability inspection method for discovering the most serious problems with a low investment of resources, while representing a high cost-benefit ratio [10]. During the heuristic evaluation, a group of usability experts examine the user interface design according to a set of usability guidelines [11]. A list of heuristic violations found in the interface design and an assessment of the severity of these problems is generated [11]. The results can be utilized as suggestions for interface refinements. This method requires less time and resources than many other usability engineering methods. Nielsen identified 10 usability heuristics as the basic characteristics of usable interfaces [4]. Research in the past has shown that usability inspection through heuristic evaluation is an effective way to uncover user interface design problems in a broad range of clinical contexts [12]. In a previous study, heuristic evaluation combined with small-scale expert assessment was examined in the context of the design and development of a Web-based telemedicine system [12]. The study found usage difficulties related to HCI problems primarily characterized by a mismatch of the designer model and the content expert model [12]. The heuristic/usage methodology provided an incremental benefit in a variety of design activities [12]. They examined a software user interface with heuristic evaluation, software guidelines, cognitive walkthrough, and usability testing and found that heuristic evaluation by several user interface specialists yielded the highest number of serious problems with the least amount of effort [12]. A single general usability expert familiar with the kind of interface being evaluated can identify about 60% of the problems [7,13]. This method was applied to support the clinical information system during a standard Call for Tender and was found to be an efficient and cost-effective approach to choose an appropriate and useful clinical information system [14]. In another study, Zhang and colleagues applied this method to evaluate patient safety with regard to the use of medical devices [15]. Heuristic evaluation through the identification of usability problems and their severities was found to be a useful, efficient, and low-cost method to evaluate patient safety features of medical devices [15].

### Overview of the Patient Education and Motivation Tool (PEMT)

PEMT is an interactive computer-based program that is being designed according to three sets of learning theories [16]: behavioral, cognitive, and humanistic. Two key ideas of behavioral theory are that learning is manifested by a change in behavior and that technology-based instructional materials should be introduced in increments. Cognitive learning theory focuses on providing structured education to individuals along with reinforcement. Humanistic theory predominantly emphasizes the participants’ willingness to learn and their ability to be evaluated. The outcome of learning depends upon how the information is presented and how the learner processes that information.

A computer-based educational program provides individuals with a self-paced learning environment and presents educational modules as a series of short messages. The information is provided in various representations, including audio, images, text, and animation with the resulting program being interactive. The system accounts for a variety of literacy levels and learning styles amongst users. Visual learners prefer seeing what they are learning, so pictures and images help them understand ideas and information better than text-based explanations [17]. Auditory learners learn best by hearing things and remember verbal instructions well, preferring someone else read the directions to them while they do the physical work or task [18]. PEMT allows users to toggle the audio on or off based on their preferences. The tool provides users with the opportunity and flexibility to navigate modules relevant to their condition by allowing them to move forward and backward at their own pace. PEMT also provides users with access to extensive information and empowers patients to obtain pertinent information about their condition. We employed usability principles when designing the user interface [19].

PEMT has three key components [20]:

1. **Screening**: PEMT allows users to enter information about their socio-demographics at their own pace, including age, gender, education, disease severity, and prior disease knowledge through a series of multiple-choice questions. No feedback is given to the individuals during this component.
2. **Learning**: The learning material is broken down into a series of educational messages with relevant audio, images, and animations as appropriate. Individuals can move forward and backward through the messages by clicking next and back buttons. The information on each screen varies in terms of the number of paragraphs, sentences, words, bulleted items, highlights, and animations.
3. **Evaluation**: The evaluation component is a post-learning questionnaire similar to that used during the screening component. Feedback is provided to the users based on their responses. Users giving correct responses receive positive prompts and encouragement while individuals giving incorrect responses are given corrective feedback and reinforcement. The goal of the evaluation component is to track the progress of individual behavior, knowledge, and disease progression over a period of time.

These three key components of PEMT make it a multifaceted tool that can be utilized to screen individuals’ demographics, health literacy, prior knowledge, attitudes, behavior, and prior use of technology. The tool has been successfully employed in different clinical settings (including emergency departments and outpatient clinics), for different conditions, including asthma [20] and influenza [21], and across different populations (including children, parents, and caregivers). In our prior study, we implemented PEMT on a touch-screen computer in a pediatric emergency department (ED). Children with asthma and their parents used the asthma education program in the ED. The results showed significant improvement in their knowledge and found PEMT to be highly acceptable [20]. In another study, we implemented PEMT in an ED and in an inner city outpatient pediatric ambulatory center (PAC) to assess and describe changes in the knowledge, attitudes, and practice regarding the influenza vaccine in participants whose children were between 6 months to 5 years of age [21]. The results of the study showed high acceptance of PEMT, and users found PEMT easy to use with no difficulties in navigating from one screen to another [21]. Users could interact with the tool on a desktop, laptop, or tablet PC using a touch screen, keyboard, and/or mouse. The system is available as a local or Web-based application.

### PEMT Hardware and Software

PEMT is implemented in an n-tier architecture, using Adobe Flash CS3 for the presentation layer, XML for content management, Microsoft.Net Framework version 2.0 with Visual Basic.Net for program logic and data flow control, and Microsoft SQL Server 2005 for data storage. Educational content elements—including text, images, thumbnails, animations, and audio—and accessibility features—including textual descriptors and closed captions—are organized using multiple XML files. The Adobe Flash layer is used to render educational content and user interface controls dynamically. User interactions with the Adobe Flash layer—including responses to questions and navigational interactions—are captured by the .Net layer and recorded in a relational structure, linked with timestamps and a unique session identifier in the MS SQL Server database. For heuristic evaluations, the software experts used the software on desktop and laptop computers running Windows XP with a minimum configuration of a Pentium 4 processor and 512MB RAM.

## Methods

 Heuristic evaluation is better if several people conduct the evaluation independent of each other [6]. Jacob Nielsen's heuristics are probably the most used usability heuristics for user interface design [6]. The evaluation is structured in terms of recognized usability principles.

The severity of a usability problem is a combination of three factors:

- The **frequency** with which the problem occurs: Is it common or rare?
- The **impact** of the problem if it occurs: Will it be easy or difficult for the users to overcome?
- The **persistence** of the problem: Is it a one-time problem that users can overcome once they know about it, or will users repeatedly be bothered by the problem?

Three usability experts (LD, KP, and LV) used Nielsen’s usability heuristics (Table 1) while reviewing the PEMT user interface and generated a list of heuristic violations. One of the usability experts was a registered nurse with 15 years of clinical and HCI experience and had conducted numerous heuristic evaluation studies (KP). One of the other experts had 12 years of professional experience in usability design and heuristic evaluation (LV), and the third expert was a PhD student in HCI with experience in doing heuristic evaluations for several studies (LD). During the evaluation, the usability experts first reviewed the user interface of PEMT independently and generated a list of heuristic violations. The usability experts then independently rated the severity of each usability violation on the following scale [6]:

0 - I don’t agree that this is a usability problem at all

1 - Cosmetic problem only: need not be fixed unless extra time is available on the project

2 - Minor usability problem: fixing this should be given low priority

3 - Major usability problem: important to fix, so should be given high priority

4 - Usability catastrophe: imperative to fix this before product can be released

In rating the problems, persistent issues with major impact on most users received the highest severity rating. The mean severity for each violation was calculated from the individual ratings. The three independent lists were combined together to generate a single list of heuristics violations, their severity ratings, and suggestions for the correction of these violations. The three usability experts (LD, KP, and LV) discussed their individual lists together, and any disagreements in assigning the severity ratings were resolved after mutual discussions. The combined list of heuristic violations was then reviewed and changes were made.

**Table 1:** Nielsen’s usability heuristics

Usability Heuristic	Description
1. Visibility of system status	The system should always keep users informed about what is going on, through appropriate feedback within reasonable time.
2. Match between system and real world	The system should speak the users' language, with words, phrases, and concepts familiar to the user, rather than system-oriented terms. Follow real-world conventions, making information appear in a natural and logical order.
3. User control and freedom	Users often choose system functions by mistake and will need a clearly marked "emergency exit" to leave the unwanted state without having to go through an extended dialogue. Support undo and redo.
4. Consistency and standards	Users should not have to wonder whether different words, situations, or actions mean the same thing. Follow platform conventions.
5. Error prevention	Even better than good error messages is a careful design which prevents a problem from occurring in the first place. Either eliminate error-prone conditions or check for them and present users with a confirmation option before they commit to the action.
6. Recognition rather than recall	Minimize the user's memory load by making objects, actions, and options visible. The user should not have to remember information from one part of the dialogue to another. Instructions for use of the system should be visible or easily retrievable whenever appropriate.
7. Flexibility and efficiency of use	Accelerators—unseen by the novice user—may often speed up the interaction for the expert user such that the system can cater to both inexperienced and experienced users. Allow users to tailor frequent actions.
8. Aesthetic and minimalist design	Dialogues should not contain information which is irrelevant or rarely needed. Every extra unit of information in a dialogue competes with the relevant units of information and diminishes their relative visibility.
9. Help users recognize, diagnose, and recover from errors	Express error messages in plain language (no codes), precisely indicate the problem, and constructively suggest a solution.
10. Help and documentation	Even though it is better if the system can be used without documentation, it may be necessary to provide help and documentation. Any such information should be easy to search, be focused on the user's task, list concrete steps to be carried out, and not be too large.

## Results

The result of the heuristic evaluation was a combined list of violations with severity ratings. The violations were sorted by heuristic and ordered from most to least severe within each heuristic category. A total of 127 violations were identified with a mean severity of 3 (range 0 - 4). The usability problems pertaining to the system function were organized by individual screens. An excerpt of the evaluation results for the user interface prototype has been presented (Table 2). Sample heuristic violations included a “lack of feedback to the user if they didn’t answer a question and tried to proceed to the next screen”, and the “inability to exit or obtain help throughout the entire program”.

**Figure 1:**
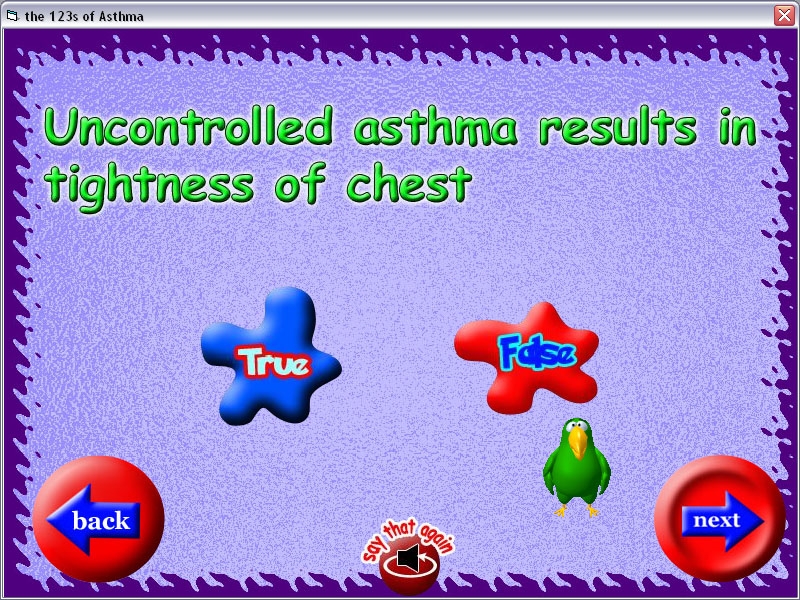
PEMT Version before heuristic evaluation

**Figure 2:**
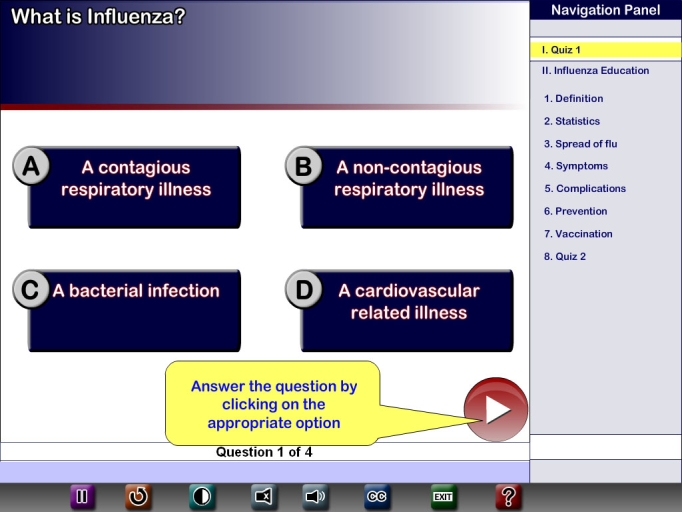
Revised PEMT version after changes were made based on heuristic evaluations

**Figure 3:**
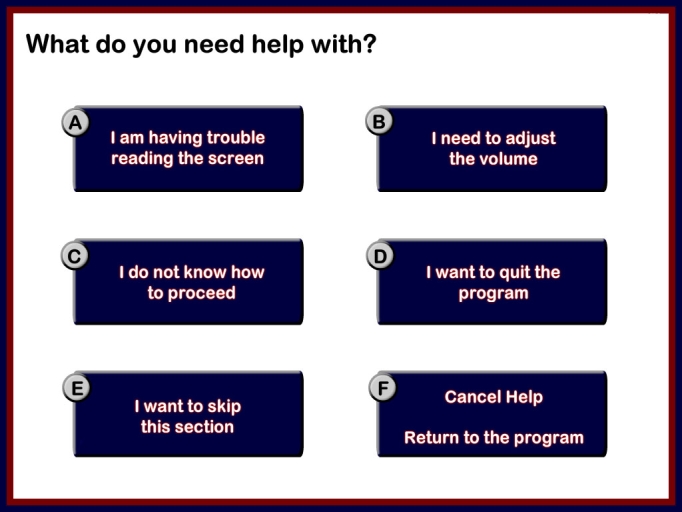
Help section is provided during the use of the program in the modified version of PEMT

The results of the heuristic evaluation were given to the software development team so that the interface could be revised. The domain expert and the software development team discussed these changes and, based on the severity ratings, changes were prioritized and implemented (Figure 1, Figure 2, Figure 3). In Figure 1, if an option is not selected and the user clicks next to go forward, no feedback is given to the user. No help is provided to assist users during their use of the program, and no exit button is available to leave the program at any time.

In Figure 2, feedback is provided if no option is selected, and the user is able to exit anytime during the use of the program.

**Table 2:** Sample heuristic evaluation results

Heuristic violated	Problem Description	Program Section	Severity Rating
Visibility	If you don't answer a question and then try to advance, the system will not let you, but it gives you no feedback on how to proceed.	Screening section	3
User control and freedom	No Exit or Quit present.	Entire program	3
Help and documentation	No Help present.	Entire program	4

Table 3 shows the average number of violations for visibility, the match between system and real world, user control and freedom, consistency and standards, error prevention, recognition rather than recall, flexibility and efficiency, aesthetic and minimalist design, help users recognize, diagnose, and recover from errors, and help and documentation. Results showed that among the 10 usability heuristics, the match between system and real world (n = 38) and consistency and standards (n = 34) were the two heuristics most frequently violated. These two heuristics accounted for more than half (57%) of all the violations. Two examples of heuristic violations related to the match between system and real world included: 1) lack of clarity in the presentation of the buttons and their functions and 2) a mismatch between the audio and written content. Consistency and standards heuristic violations included: 1) differences in function performed by similar buttons, including the “next” button that was used to display additional content on the same screen instead of to advance screens and 2) inconsistent typesizes and styles used on the same screen.

We found severity ratings predominantly higher for violations of the usability heuristics “Help users recognize, diagnose, and recover from errors” (median rating = 3) and “Help and documentation” (median rating = 4) (Table 3).

For four heuristics, more than 50% of the violations were major violations: “User control and freedom” (n = 4/6; 66.67%), “Error prevention” (n = 6/10; 54.54%), “Recognition rather than recall” (n = 1/1; 100%), and “Help users recognize, diagnose, and recover from errors” (n = 4/4; 100%) (Table 3). The median severity rating per usability heuristic has also been reported (Table 3). This information was used to evaluate the severity of the violations in each category of the usability heuristics and was used as a medium to describe not only the average number of severe violations in each category but also to prioritize changes that can be made to the violations.

**Table 3:** Number of violations, average severity rating, and severity category per usability heuristic

Usability heuristic	Median severity rating	Cosmetic	Minor	Major	Catastrophic
Visibility of system status (n = 13)	2		7	6	
Match between system and real world (n = 38)	2	15	17	6	
User control and freedom (n = 6)	3		2	4	
Consistency and standards (n = 34)	2	4	24	6	
Error prevention (n = 11)	3		4	6	1
Recognition rather than recall (n = 1)	3			1	
Flexibility and efficiency of use (n = 7)	2		4	3	
Aesthetic and minimalist design (n = 9)	2	3	6		
Help users recognize, diagnose, and recover from errors (n = 4)	3			4	
Help and documentation (n = 4)	4			1	3

Based on the feedback from the heuristic evaluations, the user interface of PEMT underwent considerable changes. The majority of the changes that required urgent attention were fixed; however, certain changes were particular to the environment in which the system was to be used which, in this case, was an emergency department setting. The changes immediately made to the system included giving users feedback in the form of a text message when they tried to navigate to the next screen without making a choice. Some of the changes that were recommended by the usability experts were not completely adopted in the revised prototype due to specific user roles and the study protocol. Designers and users are faced with different requirements and tend to focus on different sets of issues. A prior study supports the view that it is not surprising to find experts and end users faced with different requirements focusing on different sets of issues [7]. Heuristic evaluation focuses on the interface characteristics mediating between functionality and performance [7].

## Discussion

 This tutorial illustrates a heuristic evaluation using a computer-based patient education program (PEMT) as a case study. The motivation to conduct this heuristic evaluation was to uncover usability violations in the user interface prototypes of PEMT in an efficient yet effective manner. Our case study illustrates the relevance of the heuristic evaluation for identifying usability problems with computer-based health education programs. We evaluated acceptance of PEMT in the emergency department and the pediatric ambulatory clinic using an attitudinal survey. The results showed that 95% of the users found the program easy to use, 91% found it easy to navigate the program’s different screens, 94% found the text easy to read, and 93% liked the colors used on the screen [21]. Overall, the results of this study suggested high acceptance of PEMT [21]. One major weakness in our assessment of heuristic evaluation as a method is that we have no baseline against which to measure our results. Earlier studies suggest that heuristic evaluations detect 40 - 60% of the usability problems an empirical user test would find, and also claim that the types of problems found are roughly comparable [6].

A substantial benefit of heuristic evaluation is that it represents significant savings in time over the duration of a complete empirical user test, both in terms of execution and generation of interface changes for implementation. It has been reported that heuristic evaluations employing 3 - 5 evaluators can identify 60 - 70% of the usability problems in an interface, including many of the major problems, even though it requires less time than other evaluation techniques [21]. The current case study demonstrates the significance and relevance of human factors in designing computer-mediated health education programs, especially with respect to improving the acceptance of these systems.

Heuristic evaluation is a usability inspection method and differs from empirical approaches that rely heavily on user performance data, such as user testing. The study shows the practicality of heuristic evaluation. The results suggest that the application of human-computer interaction design principles to technology-based health education programs can be a quick, relatively efficient way to gather feedback and guidance to improve the interface of a system. The heuristic evaluation results were also a guide during the iterative software development process. The results were presented to the development team, and the recommended changes were implemented. The changes made to the interface were prioritized based on the severity ratings, from catastrophic to cosmetic. The immediate changes made to the program included adding a help mechanism, providing feedback to the users based on their actions, and allowing users to exit the system. Therefore, the sorted list of heuristic violations with severity ratings was very helpful for prioritizing the revisions to PEMT. The recommended changes were easily understood, not only by the software development team, but also by domain experts, since the rationale used to make changes to the system was well justified by the heuristics. The benefits of the recommended changes became evident after the revised software demonstrated higher ease of use and greater ease of navigation, while minimizing errors. Thus, providing software designers with practical feedback in a timely fashion represents a distinct advantage that heuristic evaluation possesses which many other usability engineering methods do not.

Over the long term, perhaps the most lasting result of the heuristic evaluation concerns more than the specific system tested, since heuristic evaluation also relates to internal organizational development. Heuristic evaluation was another step in this educational process. The experimenters and evaluators learned to use the method and to incorporate the results into subsequent development. A large number of usability problems were identified with a reasonable expenditure of effort. To ensure the success of education programs, information must be delivered in a way that is accessible to and meaningful to users.

However, there are several limitations of using heuristic evaluation compared to other usability engineering methods. This method relies heavily on the expertise of the usability professionals who conduct the evaluation [5]. These experts may lack domain knowledge and could therefore overlook domain-related usability problems [5]. One way to overcome this obstacle is to employ evaluators, known as double experts, who possess both usability and domain knowledge [5]. In our case study, usability experts, designers, and domain experts worked together on the design and evaluation of the PEMT. It is highly important to have a combination of these experts while evaluating computer-mediated patient education programs in the health care environment, or else there is a risk of producing a mismatch between the system and the real world. Involving professionals with expertise in both computer-mediated education and the health care environment allows for the adjustment of several variables while evaluating the system.

The PEMT user interface was more consistent with Nielsen’s usability heuristics after the expert-recommended changes were completed. In our study, we examined the value of heuristic evaluation for improving the usability of PEMT by uncovering heuristic violations in the interface design in a quick, efficient, and cost-effective manner. The ability to identify problems in a timely manner makes this method particularly well suited to the iterative design process. In addition, it is very important that the focus is on users when evaluating the interface design because this can influence the problems identified by the usability experts, as well as how these problems are described and prioritized. The system should speak the user’s language, with words, phrases, and concepts familiar to the user, rather than system-oriented terms and information, and these words, phrases, and concepts should appear in a natural and logical order. The “match between system and real world” means that the system should follow real-world conventions as closely as possible, in order to allow the user to understand how to operate the program.

We are currently conducting multiple studies to evaluate the usability of PEMT by combining heuristic evaluation and user testing for other patient education programs.

## Abbreviations

ED: emergency department
HCI: human computer interaction
PAC: pediatric ambulatory center
PEMT: Patient Education and Motivation Tool
